# Use of a Virtual Multi-Disciplinary Clinic for the Treatment of Post-COVID-19 Patients

**DOI:** 10.3390/healthcare12030376

**Published:** 2024-02-01

**Authors:** Daniella Rahamim-Cohen, Jennifer Kertes, Ilana Feldblum, Naama Shamir-Stein, Shirley Shapiro Ben David

**Affiliations:** 1 Health Division, Maccabi Healthcare Services, Tel Aviv 6812509, Israelshapira_sr@mac.org.il (S.S.B.D.); 2 Faculty of Medicine, Tel Aviv University, Tel Aviv 6997801, Israel

**Keywords:** post-COVID-19, virtual clinic, telemedicine, patient satisfaction, telehealth

## Abstract

Post-COVID-19 has been recognized as possibly affecting millions of people worldwide. In order to optimize care and ensure equality, we established a multidisciplinary virtual Post-COVID-19 clinic (VPCC) within Maccabi Healthcare Services, the second largest HMO in Israel. This study aims to describe the structure, process and patient satisfaction with this clinic. The multidisciplinary team consisted of physicians, physiotherapists, social workers, occupational therapists and dieticians. Patient entry was to be at least four weeks after COVID-19 infection. A patient satisfaction survey was carried out 7–8 months after the clinic was closed. Demographic data were collected and compared to the general Maccabi COVID-19 population. The clinic treated 1614 patients, aged 16–91, over a period of 18 months. In total, 679 family physicians referred patients. In comparison to the general COVID-19 population, a higher percentage of the VPCC patients lived in the periphery of Israel, South (14.9% compared to 17.8%) and North (17.1% compared to 18.2%). In total, 249 patients answered the survey, and of them, 75% were highly satisfied with the medical care of the physician in the VPCC. A total of 54% of respondents would have preferred a face-to-face consultation, but 50% felt that communication was good in the virtual mode. In conclusion, the VPCC provided a dedicated service for patients, and the virtual format made it equally accessible to all parts of the country.

## 1. Introduction

Since the beginning of the COVID-19 pandemic in December 2019, much has been written about the virus, disease manifestations, treatments and outcomes [1,2]. The pandemic has had a far-reaching public-health impact, both in Israel and throughout the world. It has created an unprecedented health challenge due to its significant morbidity and mortality, manifesting both in the hospital and community settings. These challenges needed to be met through coordinated efforts by healthcare systems, communities and governments that had to adapt rapidly to the evolving situation [3,4]. 

One of these challenges was the growing realization that patients were reporting various symptoms and sequelae following the acute phase. Reported symptoms may have been ongoing from the acute phase or manifested following what was considered full recovery [2,5]. Within a few months from the beginning of the COVID-19 pandemic, the Post-COVID-19 condition was recognized, and in 2021, the WHO convened a Delphi process to define and classify the condition, naming it the “post-COVID condition”; although, it is also known as “Long COVID”, “Long-Haul COVID” and other names [6]. Since then, over 50 symptoms have been reported [7] as long-term effects of COVID-19, the most common being fatigue, headache, attention disorder, hair loss and dyspnea [1,2,3,7,8]. The reported prevalence of the condition varies widely between studies [9] depending on various criteria such as patient selection, or Post-COVID-19 definition, with a meta-analysis by Chen finding a global Post-COVID-19 condition estimate of 43% [10]. Due to the uncertainty regarding the pathophysiology of Post-COVID-19, the medical complexity of patients and the minimal experience most primary care physicians have dealing with Post-COVID-19 condition patients [11], there was a need to establish specialized clinics for this emerging condition [7,9,11]. The clinics, established in countries around the world [10], functioned under various care models, most of them being multidisciplinary ambulatory-care clinics [11,12]; although, some included video consultations [12] or mobile clinics [13], as accessibility became an increasingly important factor in treatment delivery [14]. 

Various modalities have been used to provide care, including telephone consultations [15] and video [16], and for a wide range of consultation types, including dental, orthopedics [17], mental health issues [18] and many others. As technology continues to advance and healthcare systems adapt, virtual consultations are increasingly recognized as a valuable and sustainable component of healthcare delivery [14,15,16,17,18]. The utilization of digital platforms for medical consultations offers several advantages. Firstly, virtual consultations enhance accessibility to healthcare services, overcoming geographical barriers and enabling individuals to connect with healthcare professionals from the comfort of their homes. This is particularly beneficial for patients with limited mobility, those residing in remote areas, or individuals facing challenges in accessing traditional healthcare settings. Secondly, the convenience of virtual consultations contributes to heightened patient satisfaction. The flexibility of scheduling appointments, reduced waiting times, and the ability to participate in medical visits without the need for travel have been well-received by patients. 

Physicians have the ability to reach a broader patient base.

At MHS, we decided to establish a multidisciplinary virtual Post-COVID clinic (VPCC), choosing a virtual clinic model rather than an in-person one in order to enable all patients equal access regardless of their geographic location.

The primary purpose of the clinic was to address the unique needs of Post-COVID-19 condition patients and provide them with comprehensive care. The clinic had a multidisciplinary approach, including doctors, physical therapists, occupational therapists, social workers, dieticians and other specialists. This interdisciplinary team worked together to evaluate and manage the diverse symptoms and complications experienced by patients.

Virtual consultations have been shown to be effective, providing high-quality care while maintaining patient and physician satisfaction [14]. 

The aim of this study is to describe the structure, process and patient satisfaction with the Maccabi virtual Post-COVID clinic. 

## 2. Methods

This was a retrospective study, in the second-largest Health Maintenance Organization (HMO) in Israel, Maccabi Healthcare Services (MHS), serving over 2.5 million members in the community. All patients’ medical records are retained in a nationwide centralized database, spanning over 20 years, that includes comprehensive demographic and medical data for all members, including laboratory tests and hospitalization data. 

The study population included all MHS members with a positive SARS-COV-2 PCR or reported antigen test (carried out by MHS or Ministry of Health (MOH)) from March 2020 (first case in Israel) until the beginning of July 2022 (end of study period). In MHS, most of the COVID-19 patients were followed up by either a centralized, bespoke, online service [4] or by their family physicians, on the basis of an organizational scoring system of corona-risk severity [19]. The study population was then divided into two groups: those who were treated in the Post-COVID-19 clinic and those who were not. The VPCC was active from 17 January 2021 to 6 March 2022, when it closed for new referrals due to financial considerations. Data were collected until the beginning of July to include all consultations booked within the clinic format.

### 2.1. The Dedicated Post-COVID-19 Clinic

**Figure 1:**
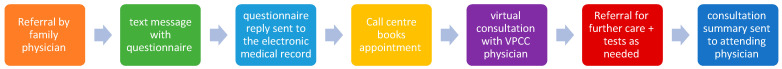
Post-COVID-19 virtual clinic patient flow.

Patients over the age of 16 years who recovered from COVID-19, 4 weeks post-recuperation could be referred to the clinic by their GP according to presenting symptoms and their clinical judgment. The CDC definition for Post-COVID, i.e., symptoms 4 weeks after the acute infection [20], was chosen. A dedicated referral form was created, which specified clinical parameters to be completed, including physical examination, vital signs (pulse, blood pressure reading, height, weight, BMI, O2 saturation and an electrocardiogram) and blood work (full blood count, urea, creatinine, liver function tests, electrolytes, ferritin, CRP and vitamin D). Once filled, the referral was electronically sent to a share point, where a Maccabi representative would contact the patient to book a virtual intake appointment with one of our specially trained VPCC GPs. Upon referral, the patient received a text message containing information about the clinic and a digitalized, detailed intake questionnaire. Questionnaire responses were entered automatically into the database and were available for the clinic physicians prior to the appointment (Figure 1).

Attending physicians were specialists in family medicine who had undergone specialized Post-COVID-19 training with the Maccabi national infectious disease specialist. Virtual consultation was carried out via telephone or video. The appointment included a comprehensive medical evaluation of the patient’s ongoing symptoms, medical history and any potential underlying conditions. In addition, the physicians would review the patients’ intake questionnaire responses. Further evaluation, referral to specialists, further testing and treatment were tailored according to each patient’s needs, ensuring an individualized approach to their recovery process. Follow-up appointments were scheduled by the physician as necessary. A clinical summary was then sent to the referring physician, detailing the evaluation and clinical recommendations. 

Consultations with the multidisciplinary team were also conducted virtually. In specific cases that required a face-to-face meeting, an appointment at one of the local Maccabi clinics would be arranged. The team consisted of experienced physiotherapists specializing in exercise counseling, who provided remote diagnosis and personalized exercise plans using training materials and videos. Occupational therapists diagnosed patients and offered cognitive strategies, fatigue management and energy conservation techniques, and interventions to improve physical function. Home safety training and fall prevention measures were also emphasized. A dietitian conducted thorough nutritional assessments and provided individualized recommendations. Social workers conducted a full intake, supported patients, addressing stress, depression and anxiety, family dynamics, and provided guidance and resources to help families navigate the challenges and maintain healthy functioning. Social workers also guided patients in negotiating their rights and entitlements to services. Multidisciplinary meetings with all clinical caregivers were carried out regularly.

### 2.2. Satisfaction Survey

Clinic satisfaction was assessed using an 8-item questionnaire, divided into two domains: feedback regarding the virtual format of the service and overall satisfaction of the VPCC. Values ranged from 1 to 7, where a value of 7 corresponds with the highest degree of satisfaction, and 1 is the lowest level. Questionnaires were conducted in Hebrew by telephone to MHS members who were patients in the clinic during the previous year. Consent was given verbally by the patient before carrying out the conversation. 

## 3. Analysis

Demographic and health data were extracted from the Maccabi database for the total study population. Demographic data included age (as of diagnosis), sex, socio-economic bracket and population group (based on home address, categorized according to Central Bureau of Statistics and Census categories). Baseline status health data included presence of selected chronic illnesses, obesity status (based on the most recent BMI record) and data specific to COVID-19 disease. Co-morbidities such as diabetes and oncology background were identified from MHS’ registries. These registries are based on validated inclusion and exclusion criteria (considering coded diagnoses, treatments and laboratory findings, as applicable). The registries are continuously and retrospectively (since 1998) updated based on each patient’s central medical record.

In addition, for all those who attended the VPCC, the referral by the VPCC physician to selected health professionals post-VPCC intake was collected.

All data were held in Maccabi healthcare services secure servers. 

Differences between the two groups (VPCC presenters and non-presenters) were evaluated with the Chi-square statistic for categorical variables and *t*-test/one-way ANOVA for continuous variables. Pearson’s correlation coefficient was used to compare correlation between continuous variables. Cronbach alpha statistic was calculated for scale items to determine cohesiveness/reliability of items. SPSS version 24 (IBM©) was used to carry out the analyses. 

The study was approved by the Maccabi Healthcare Services Helsinki committee, number MHS-20-128. Informed consent was waived by the committee.

## 4. Results

Between 17 January 2021 and 3 July 2022, 1614 patients presented to the virtual Post-COVID-19 clinic (VPCC), 1097 (68%) of whom were women. Patients’ ages ranged from 16 to 91 years (average: 45 years). From the beginning of the epidemic in Israel until the end of the study period, 839,582 MHS patients recuperated from COVID-19. In total, 679 Maccabi family physicians (of approximately 1100) referred patients to the clinic. Characteristics of recuperated COVID-19 patients by VPCC presentation status are shown in Table 1. 

Patients presenting to the VPCC were more likely to be middle-aged (35–54), female, from mid-level socio-economic neighborhoods and less likely to come from minority population groups (Table 1). In comparison to the general COVID-19 population, a higher percentage of patients attending the clinic came from the periphery: South (14.9% compared to 17.8%), North (17.1% compared to 18.2%) and Jerusalem and Plains (24.9% compared to 27.2%). Twenty-three percent of the study population had at least one chronic illness, the most common being hypertension, with those presenting to the VPCC being more likely to have a co-morbidity (Table 1). VPCC presenters were less likely to be obese (2.8%) as compared to the general COVID-19 population (8%) (Table 1). 

Those presenting to the VPCC were much more likely to have had multiple episodes of COVID-19 (24.2%) and more likely to have been hospitalized during the episode prior to VPCC entry. 

**Figure 2:**
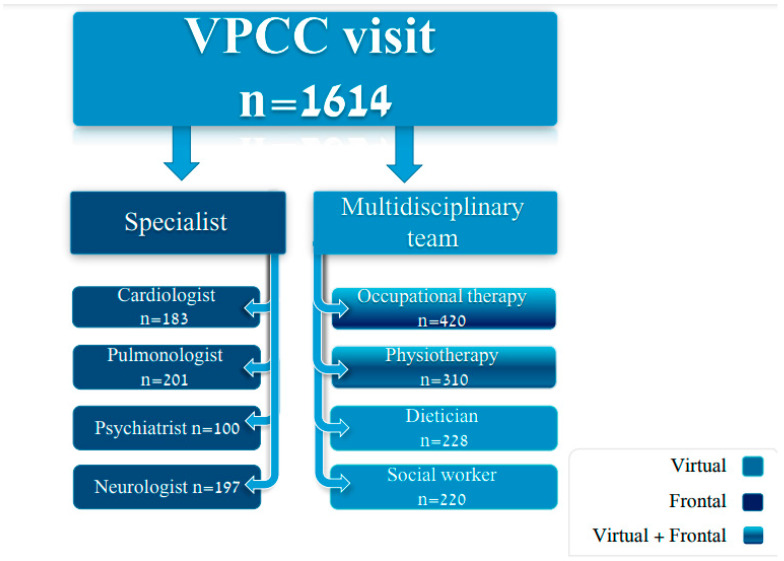
Distribution of specialist and multidisciplinary visits following a visit to the VPCC.

All 1614 patients attending the VPCC were assessed by VPCC family physicians (Figure 2). More than half of the patients, 845 (52.3%), were referred on to the dedicated multidisciplinary team: 420 (26%) were evaluated by an occupational therapist, 310 (19.2%) by physiotherapist, 228 (14.1%) by a dietician and 220 (13.6%) by a social worker. Only 523 (32.4%) patients were referred for further evaluation with a secondary care physician.

### Patient Satisfaction Survey

During February–March 2023, a telephone survey was carried out, 7–8 months following the clinic closure, and included 360 VPCC patients with a 69% (249 patients) response rate. 

**Figure 3:**
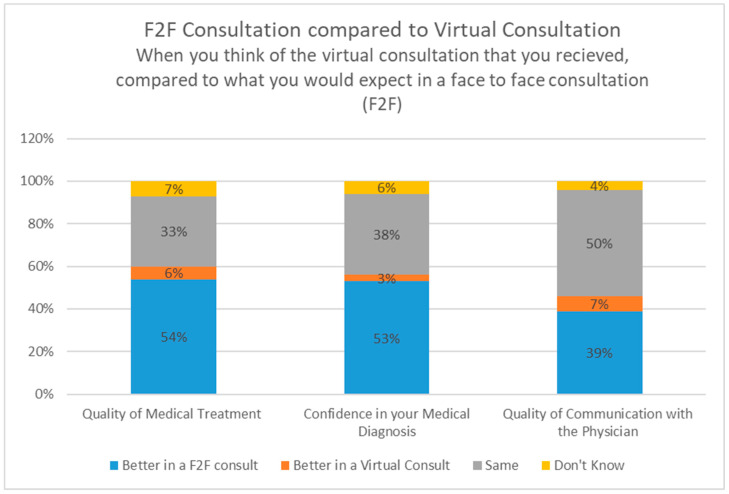
Comparison of the virtual consultation to the expectation from a face-to-face consultation.

**Figure 4:**
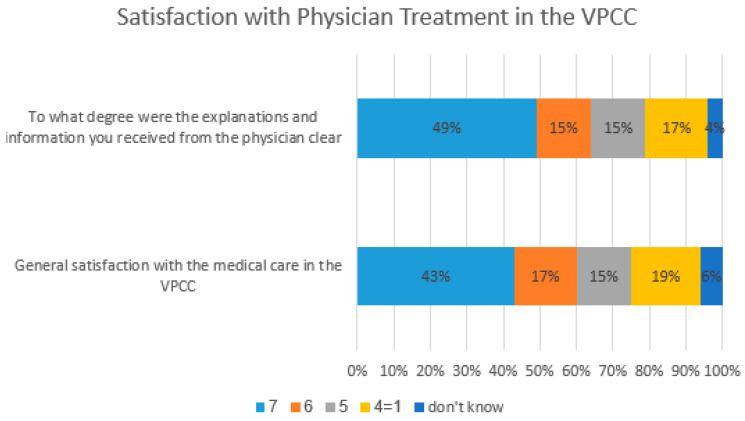
Satisfaction with physician treatment in the VPCC. Scale: 7—very high; 6—high; 5—high average; 4—average; 3—low average; 2—low; 1—very low.

Patients were asked to assess how the virtual consultation compared to what they would expect from a face-to-face consultation. In terms of quality of medical treatment, the majority of patients (54%) felt that it was better during a face-to-face consultation (F2F), with 6% preferring virtual consultation, and 33% feeling that they are the same. Results were similar for confidence in medical diagnosis, with 53% feeling that it was better in face-to-face consultations, 3% in virtual and 38% considering it the same. Quality of communication with the physician was better for 39% in face to face, 7% virtual and 50% felt it was the same (Figure 3). A total of 75% of patients gave a score of 5+ for general satisfaction with the medical care of the physician in the clinic. In total, 79% gave a score of 5+ when rating how satisfied they were with the clarity of information and explanations received (Figure 4).

**Figure 5:**
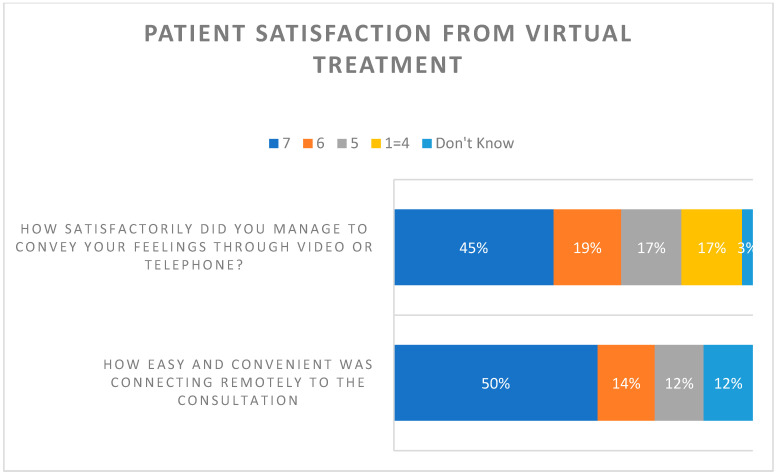
Patient satisfaction scores from the virtual treatment. Scale: 7—very high; 6—high; 5—high average; 4—average; 3—low average; 2—low; 1—very low.

Patient satisfaction with technical ease of connecting and using the virtual consultation was scored as 7 (50%), 6 (14%) and 5 (12%). The adequacy of conveying their feelings during the telephone or video consultation was scored as 7 (45%), 6 (19%) and 5 (17%) (Figure 5). 

## 5. Discussion

We established the virtual Post-COVID-19 clinic in recognition of a growing population presenting with long-term symptoms following COVID-19 infection. To our knowledge, this is the only completely virtual Post-COVID-19 clinic described. A virtual modality was chosen to facilitate access to the clinic, as people were more inclined to adopt remote consultations during the pandemic and were satisfied with the service [21,22]. The clinic was open for referral to all family physicians who felt their patients needed further attention regarding a possible diagnosis of the Post-COVID-19 condition, with minimal inclusion criteria. This was in recognition of the widespread presentation of patients with symptoms following recovery from acute COVID-19 to community physicians and the lack of experience and knowledge that these physicians had in evaluating and treating them. The clinic succeeded in addressing this need, as over half of Maccabi family doctors sent at least one patient referral to the clinic. In order to expand awareness and the knowledge base of the condition in the community, webinars and information emails were sent to all physicians updating them regarding our findings in the clinic. In addition, physicians had access to consult with a specialist in infectious diseases and with the clinic physicians via email and telephone. 

Demographic characteristics were comparable to previous studies of Post-COVID-19 [1] including a higher prevalence of females and patients in the mid-age group [23,24]. One of the main advantages was the accessibility of the clinic to all parts of the country where access equality was an important motivation to establishing the clinic. The proportion of patients from the South, Jerusalem and Plains and the North—the three areas that are considered periphery in Israel—was higher in the VPCC than in the general COVID-19 population. 

However, minority groups and patients from a lower socioeconomic bracket were under-represented among VPCC patients. This finding is consistent with other studies [21,25], indicating that these populations are less likely to use virtual medicine modalities. Both the orthodox Jewish community and the Arab community in Israel are in the lower socioeconomic brackets. It is well established that the orthodox Jewish sector does not use virtual modalities as much as other populations [26], explaining the lower percentage of patients from this community in the VPCC. However, the Arab population showed an even greater significant decrease in representation between the COVID-19 general population and the VPCC (50%); although, they do not seem to have a principle resistance to use, but rather a need for cultural and language adaptation [27]. As the majority of the Arab population has the necessary technology, there is a greater potential to engage this population with telemedicine services by recognizing and addressing cultural barriers such as women’s need for privacy in the consultation and provision of services in the Arabic language. 

In our study, 2.8% of VPCC patients were obese as compared to the general COVID-19 population (8%). This differs from the article by Vimercati et al. [28] that presented an association between Post-COVID-19 and elevated BMI. The reason for this difference is not clear; although, one possibility is that obese patients were possibly more gravely ill during COVID-19, and therefore, they were referred to hospital clinics rather than to our virtual one. 

The clinic had a significantly higher proportion of patients (8.1%) that were hospitalized during the acute phase of their illness compared to the general COVID-19 population (1.1%), consistent with the profile of Post-COVID-19 [29] patients. In addition, there was a significantly higher percentage of patients (24.4%) that had more than two episodes of COVID-19 as compared to the general COVID-19 population (6.6%). This is also consistent with the literature [1,29], as multiple COVID-19 infections have been associated with Post-COVID-19. 

The patient survey showed that the majority of patients seemed to prefer a face-to-face consultation rather than a remote one, even though half felt that there was no difference in terms of quality of the communication with the physician. This is particularly interesting and has been addressed in a study by Hawrysz [30] highlighting that most patients, given the option, would prefer a face-to-face consultation and that the high level of satisfaction does not relate to the specific service but rather to the availability of any form of healthcare. When faced with a choice, in-person consultations were also preferred by participants in a study by Predmore [31], with younger, higher-income and more-educated individuals tending to prefer remote consultations. 

The majority of patients felt that it was very easy to connect and carry out the consultation technically (very high and high—64%), and most also felt that they managed to express their concerns (64%) well through the virtual modalities; although, other factors not associated with the consultation modality and not recorded in the survey may influence this as well. Most of the patients were highly satisfied with the treatment by their clinic physician and graded the clarity and understanding of information received as high. This is in line with the literature, which cites high levels of patient satisfaction for various types of virtual clinic [32]. Although VPCC satisfaction was high regarding communication, lower scores were evidenced for diagnosis and quality of clinical treatment. One possible reason is the nature of these patient’s symptoms, many of which would have benefited from physical examination that could not take place. Data from a study by Garg [33] showed that 63%-91% of patients surveyed that attended an in-person post-COVID clinic were satisfied with clinical services, which is quite a broad range, with 71% answering they were satisfied or very satisfied with the physician interaction in the clinic, comparable to the 75% satisfaction rate with the medical care in our VPCC.

The entity of Post-COVID-19 was just beginning to be defined at the time the clinic began, and both clinicians and patients were being exposed to its nature, with pathophysiology, symptoms, evaluations and outcomes being unclear. This prevented clinicians from being able to clearly match expectations with patients regarding their condition—a factor that may have contributed to the lower confidence in diagnosis in general. More research is needed in order to evaluate this finding. Findings and analysis relating to symptoms and clinical evaluation will be presented in a following paper. 

### Limitations of the Study

The patient satisfaction questionnaire was conducted several months after the clinic closed, raising a possible recollection bias. The VPCC and surveys were conducted only in Hebrew, causing a potential barrier with non-speakers. There is no comparison with a non-virtual Post-COVID-19 clinic, which would enable a more specific analysis of the virtual modality. There is a need for long term follow-up of patients to evaluate the impact of the clinic. VPCC patients were compared to all Post-COVID-19 patients in the MHC. Temporal differences in COVID-19 prevalence and strain may have differed between the VPCC group and the remaining COVID-19 population.

## 6. Conclusions

The Post-COVID-19 virtual clinic provided a dedicated service for patients and assistance to physicians treating patients with post-COVID-19. The virtual format made the clinic equally accessible to all parts of the country and provided a highly satisfactory service to most patients. When considering future virtual clinics, attention needs to be given to possible cultural, language and technological barriers. More research into treatment delivery and follow-up needs to be conducted for Post-COVID-19 patients in order to provide an ongoing service for this new medical condition. 

## Figures and Tables

**Table 1:** Demographic and health characteristics of COVID-19-recuperated patients aged 16+ by VPCC presentation status, Maccabi HealthCare Services, July 2022. Characteristics of recuperated COVID-19 patients attending the virtual Post-COVID clinic.

Characteristic	Category	No VPCC Presentation N = 837,968	VPCC Presentation N = 1614	*p* Value
**Demographic:**				
Sex	% females	55.2	68.0	<0.001
Age group *	<25	20.2	11.0	<0.001
	25–34	19.6	18.0	
	35–44	18.3	22.3	
	45–54	19.5	27.6	
	55–64	11.7	15.1	
	65+	10.6	6.1	
Population group	General	86.0	90.0	<0.001
	Jewish Orthodox	9.0	7.4	
	Arab	5.0	2.5	
Socioeconomic status	Low	18.6	15.4	<0.001
	Middle	44.9	54.0	
	High	36.5	30.5	
Region	North	17.1	18.2	<0.001
	Sharon	21.6	20.4	
	Center	21.5	16.4	
	Jerusalem and Plains	24.9	27.2	
	South	14.9	17.8	
**Health Status**				
Heart disease *	% in registry	6.3	6.1	0.814
Diabetes *	% in registry	6.5	6.9	0.552
Hypertension *	% in registry	14.3	15.9	0.071
Cancer *	% in registry	5.4	4.1	0.020
CKD *	% in registry	7.2	7.9	0.236
COPD *	% in registry	1.1	1.7	0.035
Number of chronic illnesses **	None	76.6	74.3	0.046
	One	13.1	15.1	
	Two or more	10.3	10.7	
Obesity *	% in registry	8.0	2.8	<0.001
**COVID-19 related:**				
Number of episodes *	% with 2+	6.6	24.2	<0.001
Hospitalized during episode	% hospitalized	1.1	8.1	<0.001

* prior to/at diagnosis, BMI > 30. ** total number of chronic illnesses of (heart disease, diabetes, hypertension, cancer, CKD and COPD).

## Data Availability

Data are contained within the article.

## Abbreviations

VPCC	Virtual Post-COVID Clinic
F2F	face to face

## References

[1] Davis H.E., McCorkell L., Vogel J.M., Topol E.J. (2023). Long COVID: Major findings, mechanisms and recommendations. Nat. Rev. Microbiol..

[2] Huang C., Huang L., Wang Y., Li X., Ren L., Gu X., Kang L., Guo L., Liu M., Zhou X. (2021). 6-month consequences of COVID-19 in patients discharged from hospital: A cohort study. Lancet.

[3] Coronavirus Resource Center (2022). COVID-19 Dashboard by the Center for Systems Science and Engineering (CSSE) at Johns Hopkins University (JHU). Johns Hopkins University & Medicine. https://coronavirus.jhu.edu/map.html.

[4] David S.S.B., Cohen D., Karplus R., Irony A., Ofer-Bialer G., Potasman I., Greenfeld O., Azuri J., Ash N. (2021). COVID-19 community care in Israel—A nationwide cohort study from a large health maintenance organization. J. Public Health.

[5] Sudre C., Murray B., Varsavsky T., Graham M., Penfold R., Bowyer R. Attributes and predictors of Long-COVID: Analysis of COVID cases and their symptoms collected by the Covid Symptoms Study App. BMJ.

[6] Soriano J.B., Murthy S., Marshall J.C., Relan P., Diaz J.V., WHO Clinical Case Definition Working Group on Post-COVID-19 Condition (2022). A clinical case definition of post-COVID-19 condition by a Delphi consensus. Lancet Infect. Dis..

[7] (2020). Meeting the challenge of long, COVID. Nat. Med..

[8] Lopez-Leon S., Wegman-Ostrosky T., Perelman C., Sepulveda R., Rebolledo P.A., Cuapio A., Villapol S. (2021). More than 50 long-term effects of COVID-19: A systematic review and meta-analysis. Sci. Rep..

[9] Parotto M., Gyöngyösi M., Howe K., Myatra S.N., Ranzani O., Shankar-Hari M., Herridge M.S. (2023). Post-acute sequelae of COVID-19: Understanding and addressing the burden of multisystem manifestations. Lancet Respir. Med..

[10] Chen C., Haupert S.R., Zimmermann L., Shi X., Fritsche L.G., Mukherjee B. (2022). Global Prevalence of Post-Coronavirus Disease 2019 (COVID-19) Condition or Long COVID: A Meta-Analysis and Systematic Review. J. Infect. Dis..

[11] Décary S., De Groote W., Arienti C., Kiekens C., Boldrini P., Lazzarini S.G., Dugas M., Stefan T., Langlois L., Daigle F. (2022). Scoping review of rehabilitation care models for post COVID-19 condition. Bull. World Health Organ..

[12] Verduzco-Gutierrez M., Estores I.M., Graf M.J.P., Barshikar S., Cabrera J.A., Chang L.E., Eapen B.C., Bell K.R. (2021). Models of Care for Postacute COVID-19 Clinics: Experiences and a Practical Framework for Outpatient Physiatry Settings. Am. J. Phys. Med. Rehabil..

[13] Stallmach A., Katzer K., Besteher B., Finke K., Giszas B., Gremme Y., Abou Hamdan R., Lehmann-Pohl K., Legen M., Lewejohann J.C. (2023). Mobile primary healthcare for post-COVID patients in rural areas: A proof-of-concept study. Infection.

[14] Berlin A., Lovas M., Truong T., Melwani S., Liu J., Liu Z.A., Badzynski A., Carpenter M.B., Virtanen C., Morley L. (2021). Implementation and Outcomes of Virtual Care Across a Tertiary Cancer Center during COVID-19. JAMA Oncol..

[15] English S., Coyle L., Bradley S., Wilton W., Cordner J., Dempster R., Lindsay J.R. (2021). Virtual fracture liaison clinics in the COVID era: An initiative to maintain fracture prevention services during the pandemic associated with positive patient experience. Osteoporos. Int..

[16] Parker K., Chia M. (2021). Patient and clinician satisfaction with video consultations in dentistry—Part one: Patient satisfaction. Br. Dent. J..

[17] Sharareh B., Schwarzkopf R. (2014). Effectiveness of telemedical applications in postoperative follow-up after total joint arthroplasty. J. Arthroplast..

[18] Yuen E.K., Herbert J.D., Forman E.M., Goetter E.M., Juarascio A.S., Rabin S., Goodwin C., Bouchard S. (2013). Acceptance based behavior therapy for social anxiety disorder through videoconferencing. J. Anxiety Disord..

[19] Mizrahi Reuveni M., Kertes J., Shapiro Ben David S., Shahar A., Shamir-Stein N., Rosen K., Liran O., Bar-Yishay M., Adler L. (2023). Risk Stratification Model for Severe COVID-19 Disease: A Retrospective Cohort Study. Biomedicines.

[20] CDC. https://www.cdc.gov/coronavirus/2019-ncov/long-term-effects/index.html.

[21] Luo J., Tong L., Crotty B.H., Somai M., Taylor B., Osinski K., George B. (2021). Telemedicine Adoption during the COVID-19 Pandemic: Gaps and Inequalities. Appl. Clin. Inform..

[22] Al-Rayes S., Alumran A., Aljanoubi H., Alkaltham A., Alghamdi M., Aljabri D. (2022). Awareness and Use of Virtual Clinics following the COVID-19 Pandemic in Saudi Arabia. Healthcare.

[23] Heightman M., Prashar J., Hillman T.E., Marks M., Livingston R., Ridsdale H.A., Bell R., Zandi M., McNamara P., Chauhan A. (2021). Post-COVID-19 assessment in a specialist clinical service: A 12-month, single-centre, prospective study in 1325 individuals. BMJ Open Respir. Res..

[24] Subramanian A., Nirantharakumar K., Hughes S., Myles P., Williams T., Gokhale K.M., Taverner T., Chandan J.S., Brown K., Simms-Williams N. (2022). Symptoms and risk factors for long COVID in non-hospitalized adults. Nat. Med..

[25] Darrat I., Tam S., Boulis M., Williams A.M. (2021). Socioeconomic Disparities in Patient Use of Telehealth During the Coronavirus Disease 2019 Surge. JAMA Otolaryngol. Head Neck Surg..

[26] Latzer Y., Herman E., Ashkenazi R., Atias O., Laufer S., Biran Ovadia A., Oppenheim T., Shimoni M., Uziel M., Stein D. (2021). Virtual Online Home-Based Treatment During the COVID-19 Pandemic for Ultra-Orthodox Young Women with Eating Disorders. Front. Psychiatry.

[27] Penn N., Laron M. (2023). Use and barriers to the use of telehealth services in the Arab population in Israel: A cross sectional survey. Isr. J. Health Policy Res..

[28] Vimercati L., De Maria L., Quarato M., Caputi A., Gesualdo L., Migliore G., Cavone D., Sponselli S., Pipoli A., Inchingolo F. (2021). Association between Long COVID and Overweight/Obesity. J. Clin. Med..

[29] Pérez-González A., Araújo-Ameijeiras A., Fernández-Villar A., Crespo M., Poveda E., Cohort COVID-19 of the Galicia Sur Health Research Institute (2022). Long COVID in hospitalized and non-hospitalized patients in a large cohort in Northwest Spain, a prospective cohort study. Sci. Rep..

[30] Hawrysz L., Gierszewska G., Bitkowska A. (2021). The Research on Patient Satisfaction with Remote Healthcare Prior to and during the COVID-19 Pandemic. Int. J. Environ. Res. Public. Health..

[31] Predmore Z.S., Roth E., Breslau J., Fischer S.H., Uscher-Pines L. (2021). Assessment of Patient Preferences for Telehealth in Post–COVID-19 Pandemic Health Care. JAMA Netw. Open..

[32] Nanda M., Sharma R. (2021). A Review of Patient Satisfaction and Experience with Telemedicine: A Virtual Solution During and Beyond COVID-19 Pandemic. Telemed. J. e-Health.

[33] Garg A., Subramain M., Barlow P.B., Garvin L., Hoth K.F., Dukes K., Hoffman R.M., Comellas A.P. (2023). Patient Experiences with a Tertiary Care Post-COVID-19 Clinic. J. Patient Exp..

